# Lipid Nanoparticles for Organ-Specific mRNA Therapeutic Delivery

**DOI:** 10.3390/pharmaceutics13101675

**Published:** 2021-10-13

**Authors:** Magdalena M. Żak, Lior Zangi

**Affiliations:** 1 Cardiovascular Research Institute, Icahn School of Medicine at Mount Sinai, New York, NY 10029, USA; magdalena.zak@mssm.edu; 2 Department of Genetics and Genomic Sciences, Icahn School of Medicine at Mount Sinai, New York, NY 10029, USA; 3 Black Family Stem Cell Institute, Icahn School of Medicine at Mount Sinai, New York, NY 10029, USA

**Keywords:** modRNA, gene therapy, mRNA delivery, LNP, mRNA therapeutics

## Abstract

**Simple Summary:**

**This article belongs to the Special Issue mRNA Therapeutics: A Themed Issue in Honor of Professor Katalin Karikó**.

**Abstract:**

Advances in the using in vitro transcribed (IVT) modRNA in the past two decades, especially the tremendous recent success of mRNA vaccines against SARS-CoV-2, have brought increased attention to IVT mRNA technology. Despite its well-known use in infectious disease vaccines, IVT modRNA technology is being investigated mainly in cancer immunotherapy and protein replacement therapy, with ongoing clinical trials in both areas. One of the main barriers to progressing mRNA therapeutics to the clinic is determining how to deliver mRNA to target cells and protect it from degradation. Over the years, many different vehicles have been developed to tackle this issue. Desirable vehicles must be safe, stable and preferably organ specific for successful mRNA delivery to clinically relevant cells and tissues. In this review we discuss various mRNA delivery platforms, with particular focus on attempts to create organ-specific vehicles for therapeutic mRNA delivery.

## 1. Introduction

mRNA therapeutics are an innovative pharmaceutical technology with the capacity to create a new type of drugs that will make personalized medicine possible [1]. Currently, there are a few methods used to therapeutically manipulate protein levels in tissues, including small molecules such as statins and other inhibitors, or recombinant proteins, such an insulin. Additionally, over the last few decades there is an increase in gene therapeutic approaches being tested in clinical trials. One such approach uses micro RNA (miRs) and small interfering RNA (siRNA) as seen in the FDA-approved drug Patisiran. Another method utilizes various viral vectors such as the adeno associated viruses (AAV) for therapeutic protein expression (Table 1). However, the use of viral vectors for gene delivery in clinical setting have various limitations, therefore there is a need for a more robust gene delivery method. Because mRNA is the link between DNA and protein creation in our cells, it has been of interest to researchers in biology and medical sciences since its discovery in the early 1960s [2]. The first therapeutic use of mRNA was in the early 1990s: Wolff et al showed that mRNA can lead to functional protein translation in murine skeletal muscle, and a couple of years later, mRNA was used to treat Brattleboro rats suffering from diabetes insipidus, with partial success of injected mRNA encoding for the missing hormone arginine vasopressin (AVP) [3,4]. While early work on mRNA showed promising results, two fundamental barriers prevent mRNA therapeutics from moving forward into clinical use. The first barrier is that mRNA elicits an innate immune response. mRNA has been shown to trigger Toll-like receptors (TLRs) TLR7 and TLR8 (which recognize single-strain RNA) in the endosome and activate RIG-1 and MDA-5 receptors (leading to protein translation shutdown) in the cytoplasm [5,6,7]. The second barrier is that mRNA rapidly degrades in the body via ribonucleases (RNases) [8,9], so that exogenous mRNA in transfected cells has a very short half-life. The immune reactivity and curtailed half-life of mRNA both limit its translatability.

To overcome these barriers, two researchers, Dr. Katalin Karikó and Dr. Drew Weissman, asked the fundamental question: what mechanism is responsible for the immunological response, triggered by TLR7 and TLR8, to mRNA? Their landmark work [10]. showed that uridine, an mRNA ribonucleotide, activates the two TLRs in the endosome. Furthermore, they demonstrated that replacing uridine (U) with naturally occurring pseudouridine (Ψ) attenuates the innate immune response. This modified mRNA (modRNA), in which U is replaced with Ψ, has shown high ability to avoid cleavage by RNase and to reduce RNA-dependent protein kinase (PKR) activity [10,11]. These results indicate that modRNA has higher translation in comparison to mRNA containing either other nucleotide modifications or uridine. Our lab and others have confirmed their study and produced similar results [12,13]. Recently, modRNA has been successfully used to deliver SARS-CoV-2 Spike protein and vaccinate millions of people around the world during the COVID-19 pandemic [14,15]. New approaches seek to locally or systemically deliver modRNA *in vivo* without degrading the mRNA. In this review, we summarize different vehicles that have been used to deliver mRNA. We will cover the advantages and disadvantages of each vehicle and point out future directions in this exciting and important field.

## 2. mRNA Delivery Methods 

Since RNA was first discovered, researchers have employed many methods of delivering it to cells. Initial techniques used naked RNA, which, as mentioned above, is prone to RNase degradation and evokes a strong proinflammatory response. More sophisticated methods sought to enable cell entry and, on a systemic level, allow sufficient circulation time for the therapeutic mRNA to reach its destination and be released into target cells.

To date, lipidbased nanoparticles (LNPs) are the only RNA therapeutic carriers approved for clinical use [14,15,16]; therefore, LNPs will be the main focus of this review. However, there are other formulations used for RNA delivery, including polymers and carbohydrate polymers [17]. Gene delivery polymers contain polycations such as polyethylenimine (PEI) [18]. Due to its positive charge and abundant amines, PEI has good affinity for nucleic acids which results in a formation of complexes with a positive surface charge [19]. *In vivo*, PEI was successfully used for aerosol gene delivery into the lungs [20]. Although PEI formulations allow high transfection efficiency *in vitro* and *in vivo*, they are also significantly cytotoxic, partly because of their poor degradability, which prevents PEI-based carriers from broader use in pre-clinical and clinical settings [19,21].

Polyesters are another group of materials being used for RNA delivery. A library of 480 biodegradable polyesters was screened *in vitro* and formulations most efficiently transfected IGROV1 cells with luciferase (Luc) mRNA were subsequently tested *in vivo*. Adding pluronic F127 decreased the overall charge of the nanoparticle and increased its stability. Further, manipulating F127 content resulted in lung-specific mRNA delivery that potentially could be used to treat pulmonary disease [22].

A miniature biodegradable polymeric matrix, LOcal Drug EluteR (LODER^TM^), was developed for prolonged siRNA delivery into the pancreatic tumor environment and, combined with chemotherapy, was tested in a phase 1/2a clinical trial [23]. LODER matrix is a copolymer of poly (lactic-co-glycolic) acid (PLGA) with high molecular weight and allows slow, prolonged siRNA release in the tumor environment over several months [24].

Naturally occurring chitosan is a carbohydrate polymer that can be used for gene delivery. Chitosan features biodegradability, biocompatibility and cationic charge that allows nucleic acid binding; however, it also has limitations such as poor water solubility and limited target capability [25].

## 3. Lipid Nanoparticles (LNPs)

Lipid nanoparticles (LNPs) are spherical vesicles made of lipids. Lipids are organic, water-insoluble lipid compounds that can form defined structures such as cell membranes due to their unique features. Lipids have a hydrophilic head and a hydrophobic tail that allow LNPs to undergo self-assembly into well-defined structures, such as cell membranes [26]. LNP-RNA systems form via hydrophobic interactions in an aqueous environment combined with electrostatic interactions between negatively charged RNA and cationic or ionizable lipids [27,28]. Though LNP-RNA formulations initially used cationic lipids to allow electrostatic interactions with RNA, their toxicity instigated a gradual shift to ionizable lipids [17,29,30]. Ionizable lipids are positively charged at low pH (which allows RNA binding) and become neutral at physiological pH, a change that helps reduce the toxicity of LNP-RNA complexes *in vivo*.

**Figure 1:**
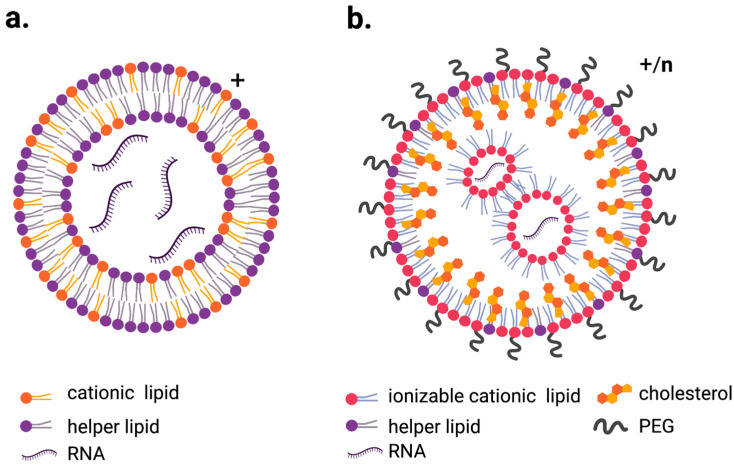
Schematic representation of the most commonly used LNPs for RNA delivery. (**a**). LNP containing cationic lipid (lipoplex) with overall positive charge, (**b**). ionizable lipid LNPs which exhibit positive charge while at low pH and more neutral charge when exposed to physiological pH. Figures created with BioRender.com.

Additional modifications tuned the properties of LNPs. For example, in addition to ionizable lipids, LNPs contain phospholipids that serve as helper lipids, cholesterol to improve cell entry and polyethylene glycol (PEG) to improve stability and circulation time by preventing serum protein binding (Figure 1a,b) [27,31,32,33].

Each LNP component can be altered to adjust the properties of the final vehicles. PEG content is inversely proportional to LNP size; changing PEG content from 1% to 5% produces LNPs 100nm to 20nm in size [34]. Similarly, raising PEG content from 0.5% to 5% results in particle sizes between 150nm and 50nm. In the same study, particles containing 0.5% PEG carrying Luc-coding mRNA showed the highest expression when injected subretinally, suggesting that particle size may be an important factor in allowing mRNA translation efficiency [35]. The primary reasons for using PEGylation in LNPs were to stabilize particles and prevent excessive serum protein binding and opsonization, which causes rapid clearance from the circulation [36,37]. However, the presence of PEG on LNP surfaces may induce anti-PEG IgM production especially after repeated administrations [38]. Because excessive immune response against PEGylated LNPs is detrimental for gene delivery, it was important to retain their stabilizing properties while avoiding anti-PEG IgM production. Manipulating PEG acryl chain length results in faster shedding from LNPs after administration. Systemic delivery of LNPs with shorter acryl chain PEGs led to lower anti-PEG IgM production following repeated administration [39]. Additionally, different naturally occurring cholesterol analogues have been shown to significantly alter LNP morphology, changes that may affect translation efficiency and thus be relevant to LNP design [40].

We will describe other application-specific modifications to LNP formulations in detail below.

### 3.1. Cationic LNPs

As mentioned above, cationic lipids are used to formulate LNPs containing nucleic acids [17]. Cationic amino groups within these lipids interact with nucleic acids’ negatively charged phosphate groups, resulting in engraftment in an LNP. In 1989, a lipoplex structure containing synthetic cationic lipid DOTMA (N-[1-(2,3-dioleyloxy)propyl]-N,N,N-trimethylammonium chloride) and helper lipid DOPE (dioleoylphosphatidylethanolamine) was used to generate Luc mRNA LNPs that successfully transfected several cell types [41]. Further, *in vitro* transfections have long used cationic lipids including commercially available Lipofectamine, which is widely used for RNA and DNA *in vitro* transfections despite its known cytotoxicity [42]. While separated, both cationic and anionic lipids in cell membranes display a cylindrical shape, which supports bilayer structure formation. However, when these lipids interact together via negatively and positively charged headgroups, they form cone-shaped structures that promote hexagonal H_II_ phase formation. This hexagonal phase disorganizes bilayer structures and correlates with membrane fusion as well as the disruption that is partially responsible for cationic lipid toxicity [43]. When systemically delivered, LNPs with permanent surface charge interact with serum proteins, and this interaction causes rapid clearance from the circulation [44,45]. Indeed, cationic LNPs have been shown to generate toxicity towards phagocytic cells *in vitro*. [46]. Additionally, systemically delivering cationic LNPs induces a strong immune response by activating the interferon type I response and instigating expression of INFγ, TNFα and the pro-inflammatory cytokine IL-2. [47]. Excessive immune reaction to LNPs is not desirable because uncontrolled cytokine release can lead to life-threating conditions; however, carefully designed immune response activation can be used as an adjuvant in RNA-LNP-based vaccines [48,49]. Though using cationic lipids has disadvantages, as detailed above, the positive charge very efficiently entraps nucleic acids. This approach lead to the development of pH-sensitive ionizable cationic LNPs for more effective RNA delivery [50].

### 3.2. Ionizable Cationic Lipids LNPs 

Currently, the LNPs that are most widely used in systemic nucleic acid delivery typically contain ionizable cationic lipids, helper (structural) phospholipids, cholesterol and PEG. Ionizable LNPs were created to avoid the toxicity of the permanently cationic lipids originally used in LNP-RNA systems, in order to enable their therapeutic applications. Patisiran (brand name Onpattro), the only FDA-approved LNP-RNA therapeutic prior to anti-SARS-CoV-2 vaccines, utilizes ionizable lipids in its LNP formulation [51]. Design of ionizable cationic lipids is balanced twofold using their p*Ka* value. The p*Ka* value is supposed to be sufficiently high so at low pH lipids are positively charged which enable binding with negatively charged RNA molecules and formation of LNPs. Thus, at low endosomal pH positive charge of ionizable lipid allow interactions with endogenous anionic lipids hence leads to disruption of endosomal structure and release of LNPs cargo into cytoplasm. Simultaneously, the p*Ka* value of the ionizable lipids should be sufficiently low so at physiological pH the surface charge of LNPs will remain relatively neutral. This allows to modulate toxicity and immunogenicity of resulting LNPs and increases their circulation time [27,40,52].

At the cellular level, the limiting factor in efficiently translating LNP-RNA is the RNA cargo release into target cells’ cytoplasm. One mechanistic explanation for the endosomal escape process is molecular structure hypothesis. According to this hypothesis, cationic ionizable lipids become protonated in increasingly acidic endosomal environments, a process that allows interaction with anionic lipids in the endosomal bilayer. That interaction forms non-bilayer hexagonal structures that disrupt the bilayer, releasing LNP cargo into cytoplasm [43,52,53]. Different cell types present varying endosomal escape mechanisms when transfected with LNP-mRNA containing cationic ionizable lipids. A study analyzing 30 different cancer cell lines concluded that the most efficiently transfecting cells exhibit rapid LNP uptake and either processing to lysosomes or rapid exocytosis. In contrast, low-transfecting cells show slower endosomal LNP trafficking to lysosomes [54]. Another study showed very low recovery of LNP-delivered mRNA in epithelial cells and determined that LNP-mRNA undergoes endocytosis and is then packed into extracellular vehicles (EV), which are subsequently secreted and detected in plasma and organs. When delivered intravenously, these EVs containing intact exogenous mRNA engendered lower levels of proinflammatory cytokines, as compared to LNP-mRNA, in mouse plasma [55]. The immunogenicity of RNA molecules might be one reason for low translation efficiency. That limitation can be resolved by using RNA containing modified nucleosides such as 1-methylpseudouridine as we previously mentioned [1,10,11].

Introducing and evolving ethanol loading procedures facilitated effective production of homogenous, small-size (diameter < 100nm) LNPs with high entrapment efficiency (>80%) [34,56,57,58]. Over the years, many ionizable lipids have been developed, with a variety of features depending on the desired purpose.

Prior to developing SARS-CoV-2 vaccines, both BioNTech and Moderna worked on LNP-encapsulated mRNA therapeutics with a range of properties optimized for different aims [59,60,61,62,63,64,65,66]. In a study published in 2015, LNP containing an ionizable cationic lipid carrying Luc modRNA was injected *in vivo* using six different delivery routes (intradermally, intramuscularly, subcutaneously, intraperitoneally, intravenously and intratracheally). The LNPs were 70-100nm in size and comprised cationic ionizable lipid, phospholipid, cholesterol and PEG at 50:10:38.5:1.5 mol/mol ratio. Expression kinetics analysis showed different expression patterns depending on the delivery route. Local intramuscular injection produced Luc expression at the injection site as well as diffusion to the liver. Additionally, systemic delivery via intravenous or intraperitoneal injection also resulted in strong protein production in the liver [67]. These findings suggest it will be important to tune LNP properties to enable the highest expression rates in the targeted tissue, according to the therapeutic goals.

Research published in 2019 compared various LNP formulations for intramuscular administration of mRNA vaccines. As noted above, in LNPs ionizable lipids’ properties depend on pH, and their p*K*a value defines their behavior at various pH levels. This study tested LNPs containing ionizable lipids with different p*K*a and found that with regard to protein expression, the best lipids for intramuscular administration have higher p*K*a than those that are best for IV administration, a result that suggests LNPs should be specifically designed their intended purposes [68]. For example, vaccines must boost innate immune stimulation with good tolerability. Of the lipid formulations tested, the authors concluded that the optimal p*K*a for immunogenicity was between 6.6 and 6.8; however, an ionizable lipids’s p*K*a is not the only factor that plays a role in optimization. Another 2019 publication focused on designing ionizable lipids for LNP formulations intended to serve as antigen mRNA delivery vehicles as well as adjuvants. This extensive study prepared a library of over 1000 lipid formulations and tested their ability to cause antigen protein expression and induce immune response for anti-tumor vaccines. The authors concluded that the formulations containing ionizable lipids with cyclic amine head groups, unsaturated lipid tails and dihydroimidazole linkers most efficiently inhibited tumor growth and increased survival in both melanoma and human papillomavirus E7 mouse models [69].

To date, ionizable lipid LNPs are the preferred carriers for clinical therapeutic RNA delivery. The anti-SARS-Cov-2 modRNA vaccines are the most prominent example, with millions of doses already administered worldwide [14,15]. Additionally, a previously mentioned Patisiran, carrying an siRNA targeting 3′ untranslated region of transthyretin mRNA was FDA-approved treatment for hereditary transthyretin amyloidosis in 2018 [16].

Having proved that LNP-RNA therapeutics are safe and efficient, the next step for the field is to focus on designing cell- and organ-specific treatments for minimally invasive clinical delivery routes. Indeed, several trials have been already completed in pursuit of these goals, and many more are ongoing.

### 3.3. Organ-Specific LNPs

The abovementioned vaccine studies used intramuscular injections of LNP-RNA for systemic immune response, as well as systemic delivery to the liver in the case of Patisiran, but how could we direct LNP-RNA into other organs for protein replacement therapy? Apolipoprotein E (ApoE) in blood serum has been shown to bind to intravenously injected LNPs. Crucial to transporting and metabolizing lipids, ApoE regulates lipoprotein and cholesterol levels in the plasma via high-affinity binding to the family of LDL receptors [70,71]. The liver is the main organ for clearing ApoE-binding lipoproteins; hence, systemically delivered LNPs would bind ApoE and preferentially home to the liver [36,72]. A study performed on *apoE*^−/−^ mice using cationic and ionizable lipid LNPs carrying siRNA demonstrated that the hepatic uptake of ionizable, but not cationic, LNPs is ApoE dependent, suggesting that LNP charge plays a role in LNP tissue tropism [73]. 

While excessive liver homing is a notable disadvantage of intravenously injected ionizable LNPs, a selective organ targeting (SORT) strategy could overcome this issue [74]. In this system, various lipid classes were designed for tissue-specific gene delivery and editing using CRISPR-Cas technology. Based on previous work, researchers speculated that the key to organ-specific delivery would be manipulating the internal and/or external charge of formulated LNPs [75,76,77]. Along with standard LNP components including an ionizable cationic lipid, phospholipids, cholesterol and PEG, the authors proposed adding SORT molecules which allow lung-, spleen- or liver-specific gene delivery. Indeed, adding increasingly higher percentages of permanently positively charged 1,2-dioleoyl-3-trimethylammonium-propane lipid (DOTAP) shifted tissue tropism from the liver to the lungs. Based on that outcome, researchers tested numerous other molecules with different charges, including a negatively charged 1,2-dioleoyl-sn-glycero-3-phosphate (18PA) SORT molecule that, at 10-40% incorporation in an LNP formulation, resulted in spleen-specific Luc expression. Incorporating SORT molecules, including DLin-MC3-DMA which was used in Patisiran [51], into several classes of ionizable LNPs produced similar outcomes, indicating this system is compatible with commonly used ionizable lipid-LNPs and can be altered according to therapeutic goals [74]. These same authors recently published another study, where they created multi-tailed ionizable phospholipids (iPhos) that facilitated endosomal release of RNA cargo. These lipids can function together with additional variously charged helper lipids to allow organ-specific delivery, an approach similar to the SORT system [78].

In addition to organ-specific delivery, researchers have pursued targeting specific cell subsets in the liver by engineering ionizable lipid nanoparticles for selective RNA delivery into hepatocytes and liver sinusoidal endothelial cells (LSEC). For hepatocyte-specific delivery, the authors manipulated particle size by adjusting PEG content, whereas incorporating mannose favored targeted LSEC RNA delivery [79].

One of the most therapeutically important targets for gene delivery is tumor tissue. Cancer remains the main cause of death worldwide, accounting for nearly 10 million deaths in 2020 [80] and various gene therapy applications, including RNA-based approaches [81], have been employed for cancer treatment in pre-clinical trials. However, as with traditional chemotherapy, the main concern remains targeted delivery to tumor tissue. To meet this challenge, a LNP-mRNA delivery system was designed to deliver CRISPR components (cLNP) into glioblastoma and disseminated ovarian tumors [82]. LNPs are typically constructed using ionizable cationic lipids; however, this study designed a library of novel-class ionizable amino lipids and compared them to the clinically used Dlin-MC3-DMA LNP formulation containing ionizable cationic lipid. In a glioblastoma model, local, intratumoral cLNP delivery inhibited tumor growth and increased survival, results that show its efficiency in PLK1 (polo-like kinase) gene editing. Because PLK1 is an enzyme involved in the cell cycle, its inhibition causes cell cycle arrest and death of dividing cells. Even though intratumor injection beneficially affected designed LNPs, the most promising path to potential clinical use is to develop tissue-targeted LNPs suitable for minimally invasive delivery methods. In pursuit of this goal, the authors created LNPs coated with cell-targeting antibodies, a system called ASSET [83]. Intraperitoneally delivering targeted LNPs against OV8 peritoneal xenografts limited tumor growth and improved survival in tumor-bearing mice. As OV8 tumor cells overexpress epidermal growth factor receptor (EGFR), the targeted LNPs contained anti-EGFR antibody [84]. However, the limitation of this approach is that only some cancer cells exhibit a distinct expression profile on their surfaces that can be targeted using antibodies. Another approach to delivering LNP-mRNA to tumors and imaging them *in vivo* deployed theranostic LNPs containing PEGylated BODIPY dyes (PBD) [85], which structurally similar to the PEGs traditionally used in LNPs. Analysis of the novel LNP library revealed that the intravenously administered 4A3-SC8&PEG2k5d formulation was preferentially expressed in liver and subcutaneous tumors. Yet this formulation contains pH-activable dye that allows high tumor-to-liver contrast fluorescence, as the authors themselves noted [86,87]. Therefore, the high signal observed in tumors might be a product of the low-pH tumor environment and not necessarily LNP accumulation in the tumor tissue. 

So far, systemic delivery into tumors remains an unmet challenge, though many researchers are exploring the use of lipoplexes, which are cationic liposomes, to carry nucleic acid as another approach to cancer immunotherapy and cancer vaccines. In a study that used RNA-lipoplexes (RNA-LPX) to convey cancer antigens into dendritic cells [75], the authors manipulated lipid to RNA ratios and compared Luc expression in different organs after systemic delivery. Decreasing cationic lipid content in RNA-LPX formulations resulted in a predominant signal in the spleen. Most importantly, in CD11c-DTR mice, which allow CD11c^+^ cell depletion, the spleen signal was not detectable after Luc-LPX administration, indicating that this formulation preferentially targeted antigen-presenting cells (APC). More detailed analysis showed that dendritic cells showed the highest protein expression even though macrophages internalized more RNA from injected RNA-LPXs. When treated with RNA-LPX bearing influenza virus hemagglutinin, both cell types induced TLR7-dependent overexpression of INFα. Systemic delivery of RNA-LPX vaccines carrying tumor-specific antigens (OVA for B16-OVA melanoma and gp70 for CT26 colon carcinoma), produced complete, long-lasting protection against subcutaneous tumor challenge, and re-challenge in the case of CT26. Additionally, RNA-LPX vaccines protected the mice from lung metastasis when delivered after intravenous tumor implantation and significantly increased their survival. The authors concluded that the observed tumor rejection occurred via INFα-dependent T-cell activation [75]. 

The formulation used in this study was also employed in a project focusing on immune system desensitization in multiple sclerosis [88]. Antigen-specific tolerization is a promising approach for treating autoimmune diseases without impairing the immune system’s primary functions [89]. In this study, the authors used nucleoside-modified mRNA with 1-methylpseudouridine (m1Ψ) to avoid TRL7-dependent INFα release by APCs caused by single-strain RNA stimulation [10,11,90]. Systemic delivery of m1Ψ-RNA-LPX coding for disease-related autoantigens generated improvements in several mouse models of multiple sclerosis. Contrary to the RNA-LPX study, m1Ψ-RNA-LPX caused neither INFα secretion nor significant APC activation, and the authors concluded that the beneficial effect was related to reduced effector T-cell levels and elevated regulatory T-cell populations. Importantly, the authors showed that Luc expression in the spleen was much higher and more prolonged when Luc was delivered using m1Ψ-RNA-LPX rather than RNA-LPX, suggesting that not only the vehicle but also RNA composition itself has tremendous impact on treatment outcome. 

Another study utilizing modified mRNA LNPs investigated therapeutic delivery of interleukin 10 (IL-10) into Ly6C^+^ inflammatory leukocytes in a mouse model of inflammatory bowel disease (IBD) [91]. In addition to using modified RNA for prolonged, robust therapeutic protein expression, researchers achieved cell specificity with an LNP formulation utilizing the previously described ASSET system which coats LNP surface with cell-specific antibodies. Here the authors used anti-Ly6c antibody and showed that systemic delivery of anti-inflammatory IL-10 into Ly6C^+^ leukocytes significantly reduced the severity of intestinal inflammation in treated mice [91].

Thoughtfully designing and tuning LNP properties is one approach for tissue-specific delivery of therapeutic RNA. Another possibility would be to create a ‘self-controlling’ RNA expression system that only allows protein translation in specific cell subsets despite RNA uptake by other cell types. The Specific Modified mRNA Translation System (SMRTs) was designed to permit specific expression of therapeutic genes in cardiomyocytes (CM) but not in non-CMs in the heart [92]. This system takes advantage of the CM-specific micro RNAs (miRs) miR-1 and miR-208 [93,94]. SMRTs is an on/off system that contains two modRNA molecules which together create a circuit; the first contains a L7AE gene and a CM-specific miR recognition element; the second contains a gene of interest and a L7AE-recognition element, a K-motif. This design allows expression of the gene of interest in CM and prevents expression in other cell types. 

## 4. Conclusions and Future Directions

Until recently, LNPs were mostly designed for small interference RNAs (siRNAs), which are significantly smaller than mRNA. More tailored LNP formulations may therefore need to be developed to allow such a big molecule to be carried and translated efficiently [95,96,97].

**Figure 2:**
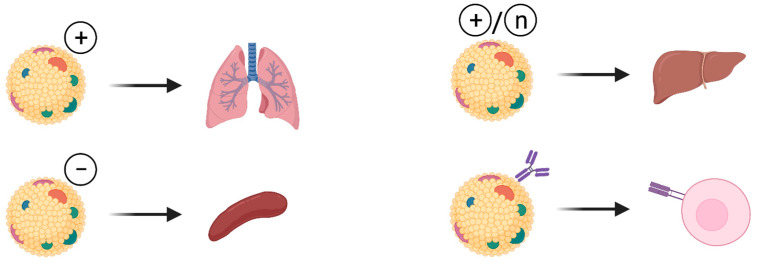
Approaches for organ specific modifications of LNPs used for RNA delivery. Attempts for organ specific modifications of LNPs include manipulation of LNP charge that promotes lung-, spleen- or liver- specific delivery of therapeutic RNA. Attaching cell specific antibody on the surface of LNP allows delivery of the RNA into target cells. Figures created with BioRender.com.

To date, the only broadly used organ-specific delivery approach is based on the overall charge of the LNPs (Figure 2). Positively charged mRNA-LNPs translate mostly, but not specifically, in the lung, neutral charge leads to expression in the liver and a negative charge allows expression in the spleen. This approach still needs improvements to prevent leakage to other organs in order to achieve true organ-specific expression of the therapeutic transgenes. Moreover, approaches to deliver LNP-RNA therapeutics to other organs need to be explored.

Tumor targeting can be improved by adding tumor-specific antibodies to the surface of LNPs. However, due to the lack of cancer-specific surface markers, this approach cannot be widely utilized. One attempt to boost tumor-specific RNA-LNP delivery is to increase its circulation time in order to expand its ability to accumulate in tumor tissue. PEGylation is one method that can promote LNP accumulation in tumor tissue after systemic delivery. Adding PEG to the LNP surface prevents protein binding and opsonization which increase circulation time but may also be related to the more favorable nanoparticle size that helps extravasation into tumor tissue [98]. Another direction for designing more effective tumor-specific RNA-LNPs is to employ unique tumor environment characteristics. Since solid tumors have lower pH than surrounding tissues [99,100], for example, incorporating pH-responsive systems into RNA-LNPs may help tumor-specific delivery [101].

In last decade, lipid-based carriers for RNA delivery received significantly increased attention in this rapidly growing field. LNPs used in mRNA vaccines against SARS-Cov2 are a safe and efficient vehicle for therapeutic gene delivery, and their success will certainly support the development of LNPs for other applications, such as protein replacement therapies or gene editing, in the future. 

## Figures and Tables

**Table 1:** Tools for therapeutic manipulation of protein levels in tissues.

	AAV	Modified mRNA	miRNA/siRNA	Small Molecules	Protein
	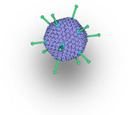	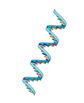	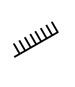	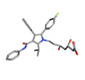	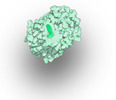
**Route of Administration**	Local or I.V	Local or I.V	Local or I.V	I.V or oral	Local
**Limitation of Gene Size**	Yes (4.5 Kb)	No	N/A	N/A	No
**Pharmacokinetics**	Long term	Short term	Short term	Long or short term	Short term
**Multiple Administration**	No	Yes	Yes	Yes	Yes
**Compromised DNA** **Integrity**	Yes	No	No	No	No
**Controlled Expression**	No	Yes	Yes	Yes	Yes
**Gene Expression** **Regulation**	Up or down	Up or down	Mostly down	Mostly down	Mostly up
**Organ Specificity**	Yes	No/possibly	No	No	No
**Cell Specificity**	Yes	No/possibly	No	No	No
